# Cytocompatibility and Osteoinductive Properties of Collagen-Fibronectin Hydrogel Impregnated with siRNA Targeting Glycogen Synthase Kinase 3β: In Vitro Study

**DOI:** 10.3390/biomedicines11092363

**Published:** 2023-08-24

**Authors:** Elena V. Galitsyna, Anastasiia A. Buianova, Vadim I. Kozhukhov, Sergey P. Domogatsky, Tatiana B. Bukharova, Dmitriy V. Goldshtein

**Affiliations:** 1 Research Centre for Medical Genetics, 1 Moskvorechye Str., 115522 Moscow, Russia; 2 Federal State Budgetary Institution, National Medical Research Centre of Cardiology, Ministry of Health of the Russian Federation, 121552 Moscow, Russia

**Keywords:** GSK3β, MSCs, siRNA, PEI, osteogenic differentiation, collagen–fibronectin hydrogel

## Abstract

In this study, we developed an osteoplastic material based on collagen–fibronectin hydrogel impregnated with siRNA molecules targeting glycogen synthase kinase 3β (GSK3β), which inhibits the osteogenic differentiation of mesenchymal stem cells. The hydrogel impregnated with polyplexes containing siRNA GSK3β and polyethylenimine has been shown to have no cytotoxic effect: there was no statistically significant change in the cell’s viability after 7 days of incubation in its presence compared to the control group. On days 2 and 7, an increase in the level of expression of markers of osteogenic differentiation was observed, which confirms the osteoinductive qualities of the material. It has been demonstrated that the hydrogel maintains cell adhesion. Our results obtained in vitro indicate cytocompatibility and osteoinductive properties of collagen–fibronectin hydrogel impregnated with siRNA GSK3β molecules.

## 1. Introduction

Glycogen synthase kinase-3β (GSK3β) is one of the regulators of osteogenic differentiation that inhibits signaling of the its main pathways, BMP and WNT/β-catenin, by phosphorylating Smad1 and β-catenin molecules and inducing their subsequent ubiquitination and proteasomal degradation [1,2]. Furthermore, GSK3β can directly phosphorylate the transcriptional factor that is responsible for the induction and completion of osteogenic differentiation, Runx2, and suppresses its transcriptional activity [3].

Small molecules are often used as *GSK3β* inhibitors due to their relatively small size. Lithium chloride (LiCl) was the first *GSK3β* inhibitor discovered and is still actively used in many studies. However, this molecule has several other biological targets that lead to undesirable phenomena and a fairly small therapeutic window (dose range, between efficacy and toxicity). Most of the other small molecules are patented or are at the stage of preclinical studies. In addition, natural ways of regulating the activity of GSK3β in human cells using the mechanism of RNA interference are known.

microRNA molecules participate in the positive and negative regulation of osteogenesis at all stages of signal transmission, affecting the mRNA of ligands, receptors, signaling molecules and transcription regulators involved in the implementation of cell differentiation. The use of microRNA-590-5p mimics and leads to the degradation of the *SMAD7* gene transcript. microRNA-29a, directed at *DKK1*, *Kremen2* and *sFRP2*; microRNA-218, the targeted genes of which are *Dkk2*, *sFRP2* and *Sost*, as well as microRNA-335-5p, targeted at *DKK1*, contribute to osteogenic differentiation in vitro [4,5,6,7,8]. Similarly, the introduction of exogenous siRNA molecules into cells directed at osteogenesis inhibitor genes—*PPAR*-*γ*, *Noggin*, *GNAS1*, *Chordin*, *GSK3β*, B-cell lymphoma (Bcl)-like 2 (*BCL2L2*), and also genes involved in bone resorption processes—*RANK*, *Plekho1* or Semaphorin 4d (*Sema4d*), promotes the osteogenic differentiation in in vitro or in vivo models [9,10,11]. 

However, the regulation of signaling pathways by means of the above-listed small molecules or microRNA molecules can lead to pleiotropic effects, since such compounds do not have a high selectivity and may have several targets at once [12,13,14]. These effects are nearly eliminated when using siRNA molecules [15], which are a highly accurate tool for regulating gene expression at the post-transcriptional level.

In this work, an approach based on decreasing the *GSK3β* gene expression at the mRNA level using siRNA molecules in order to increase the efficiency of osteogenic differentiation of mesenchymal precursors was offered for the first time. For this purpose, it is proposed to impregnate siRNA GSK3β molecules into a biocompatible polymer material for a targeted delivery to the bone defect area and prolonged release to ensure the long-term inhibition of the target gene expression.

Collagen-fibronectin hydrogel was chosen as a promising carrier matrix for siRNA molecules. Xenogeneic collagen materials have a high biocompatibility, as opposed to materials based on other components of the extracellular matrix (ECM), such as hyaluronic acid, chondroitin sulfates and elastin [16].

The aim of this investigation was to study the cytocompatibility and osteoinductive properties of the collagen–fibronectin hydrogel impregnated with siRNA GSK3β molecules to obtain an effective osteoplastic material.

## 2. Materials and Methods

### 2.1. siRNA Design

For the purpose of the knockdown of *GSK3β* mRNA using a siRNAfit program [17], developed by the Laboratory of functional genomics of the RCMG, the siRNA sequence, targeting exons 8 of the gene, was selected (Table 1). The sequence of the non-targeting siRNA molecules, bearing the fluorescent label 6-carboxyfluorescein (siRNA-C) or biotin (siRNA-B), was borrowed from the work of Horova V. et al. [18]. The siRNA molecules were manufactured in “DNA-Synthesis LLC” (Moscow, Russia).

### 2.2. Polyplexes Containing siRNA and PEI Preparation

Polyplexes, containing siRNA and linear polyethylenimine (PEI) with a molecular weight of 25 kDa, pH = 4.5 (23966-1; Polyscience, Warrington, PA, USA) in a 1:3 ratio (µg siRNA: µg transfection agent) were formed in Dulbecco’s phosphate-buffered saline (DPBS) (Gibco, Miami, FL, USA) according to the manufacturer’s instructions for 35–40 min at 37 °С (Table 2).

### 2.3. Fabrication of Collagen–Fibronectin Hydrogel Impregnated with Polyplexes, Containing siRNA GSK3β Molecules

To obtain one sample of the material, a sterile neutral 10% porcine collagen type I solution at a concentration of 40 mg/mL (Viscoli^®^, cat.PA100, “Imtek Ltd.”, Moscow, Russia) was mixed with human fibronectin (cat. H Fne-C, “Imtek Ltd.”, Moscow, Russia) in a ratio of 1:3 by volume to obtain 100 μL. To impart osteoinductive properties to the material, polyplexes consisting of 300 pmol (1.5 pmol/μL) or 600 pmol (3 pmol/μL) siRNA GSK3β and PEI in a ratio of 1:3 (µg siRNA: µg PEI) in 50 μL DPBS (“PanEco”, Moscow, Russia) were added to the collagen–fibronectin mixture. A polymerization of hydrogels was performed for 2 h at 37 °C after the addition of 50 µL buffer ANB-12 pH = 11 (“Imtek Ltd.”, Russia) to obtain 200 μL/sample. Finished samples of materials were stored at 4 °C for no more than 24 h.

### 2.4. siRNA GSK3β Molecules’ Release Monitoring

A total of 60 pmol (0.3 pmol/µL) siRNA-B or polyplexes containing 60 pmol (0.3 pmol/µL) siRNA-B and PEI in a ratio of 1:3 (µg siRNA: µg PEI) were impregnated into collagen–fibronectin hydrogel at the gelation stage according to the procedure indicated in clause 2.3. The prepared hydrogels were placed in 96-well plates and infused with 200 µL of PBS-Tween (“Imtek Ltd.”, Moscow, Russia) for 1 h, changing the solution for 3 h. Next, the solution, presumably containing siRNA-B, was incubated with 1.88 µg/mL of streptavidin conjugated with siRNA-B peroxidase for 15 min to form a biotin–streptavidin–peroxidase complex. The detection of the biotin–streptavidin–peroxidase complex was carried out by ELISA. The results were analyzed using an “EnSpire Multimode Plate Reader” (“PerkinElmer”, Waltham, MA, USA).

### 2.5. Cell Cultures

The human mesenchymal stem cells derived from human adipose tissue (hAD-MSCs) cultures at passages 2–3 were used in this work. hAD-MSCs cultures were obtained from tissues of healthy donors who signed an informed consent to participate in this study.

The cells were cultured in a growth medium of Dulbecco’s modified Eagle’s medium (DMEM) (“PanEco”, Moscow, Russia) with 10% fetal bovine serum (FBS) (“PAA Laboratories”, Dartmouth, MA, USA), 100 mg/L amikacin (“Synthesis”, Saint Petersburg, Russia), 4 mM L-glutamine (“PanEco”, Moscow, Russia), 10 µg/L FGF-2 (“ProSpec”, Saint Louis, MI, USA) and 2000 IU/L heparin sodium (“B. Braun Medical Inc.”, Melsungen, Germany) at 37 °С and 5% СО_2_. The culture medium was changed every 3 days.

### 2.6. Immunophenotyping of hAD-MSCs

Immunophenotyping of three hAD-MSCs cultures was performed to confirm the cellular phenotype. Immunophenotyping was performed using antibodies to a positive cell-surface markers of MSCs: CD29+ (ab52971; “Abcam”, Waltham, MA, USA), CD44+ (ab6124; “Abcam”, USA), CD90+ (ab23894; “Abcam”, USA), and also “Human Mesenchymal Stem Cell Multi-Color Flow Kit” (FMC020; “R and D system”, Minneapolis, MN, USA), including antibodies to positive and negative markers labeled with fluorescent dyes (Table A1). To detect fluorescence, antibodies to CD29+, CD44+ and CD90+ markers were incubated with secondary antibodies conjugated with the fluorescent dyes Alexa Fluor 488 (ab150077; “Abcam”, USA) or Alexa Fluor 555 (ab150114; “Abcam”, USA). Staining of the hAD-MSCs with antibodies was performed according to the manufacturers’ recommendations. At least 8000 cells were analyzed during the detection of each marker. The results were evaluated using flow cytometers CyFlow^®^ Space (“Partec GmbH”, Münster, Germany) and “CytoFLEX S” (“Beckman Coulter”, Brea, CA, USA), and packages “FloMax^®^ Software v2.3” (“Partec GmbH”, Münster, Germany) and “CytExpert Software v2.0” (“Beckman Coulter”, Brea, CA, USA), respectively. Histogram overlays were performed with FlowJo™ Software v10.7.1 (“FlowJo LLC”, Ashland, OR, USA).

### 2.7. Assessment of Delivery Efficiency to the hAD-MSCs Cultures

A total of 300 (1.5 pmol/μL) or 600 pmol (3 pmol/μL) siRNA-C as part of polyplexes, consisting of PEI in a ratio of 1:3 (µg siRNA: µg PEI), were added to the prepared collagen solution. Next, the mixture of collagen with siRNA were incubated in an atmosphere of ammonia vapor for 1 h. Upon completion of the polymerization process, the materials were rinsed with sterile distilled water and incubated with cells when the culture reached a monolayer confluency of 70–80% in Transwell^®^ 24-well permeable support with a pore diameter of 8 microns (“Corning Life Sciences”, Kennebunk, ME, USA) in 1 mL of Opti-MEM medium with 5% FBS (PAA Laboratories, USA) for 24 h. As a negative control, hAD-MSCs were used, which were cultured in the equivalent to experimental groups’ amounts of Opti-MEM medium with 5% FBS.

The efficiency of the cell transfection in suspension was evaluated using CyFlow^®^ Space (“Partec GmbH”, Münster, Germany) flow cytometer and FloMax^®^ Software v2.3 (“Partec GmbH”, Münster, Germany) package. Files were concatenated and analyzed in FlowJo™ Software v10.7.1 (“FlowJo LLC”, Ashland, OR, USA).

### 2.8. MTT Assay

The cytotoxicity of collagen–fibronectin hydrogel, separately and impregnated with polyplexes containing 1.5 pmol/µL siRNA GSK3β on days 1, 4 and 7 after transfection, was examined using MTT assay [19] by quantifying living cells. МТТ (“PanEco”, Moscow, Russia) was added for cell staining in plate wells at a concentration of 0.5 mg/mL and incubated for 2.5 h at 37 °C. The amount of formazan formed was determined by measuring, with an EnSpire^®^ Multimode Plate Reader (PerkinElmer, USA), the absorbance at 570 nm; the background value at 620 nm was subtracted.

### 2.9. Staining of Cells Adhered to the Surface of Materials

To assess the ability of the material to maintain cell adhesion, hAD-MSC cultures were incubated in the presence of collagen–fibronectin hydrogel separately, and they were impregnated with 1.5 pmol/µL siRNA GSK3β and PEI in a ratio of 1:3 (µg siRNA: µg PEI) for 24 h.

To visualize the total number of cells on the surface of the materials, they were pre-stained with a vital red-fluorescent dye PKH26 (PKH26GL, “Sigma-Aldrich”, St. Louis, MI, USA) for general cell membrane labeling according to the manufacturer’s instructions. A suspension of labeled cells was applied to samples of the material at a concentration of 1 million/mL and incubated for an hour in a 24-well plate in 100 µL FBS (“PAA Laboratories”, USA) for their attachment. Then, Opti-Mem medium with 5% FBS up to 1 mL was added to the wells and incubated under standard culture conditions. As a negative control, plate wells without the material were used. After 24 h, the medium for cell cultures was replaced with DMEM (“PanEco”, Moscow, Russia), containing 4 mM L-glutamine (“PanEco”, Moscow, Russia), 10% FBS, 100 mg/L amikacin (“Synthesis”, Russia).

To visualize living and dead cells after 1 and 3 days of incubation, cells on the surface of materials were stained with Calcein AM dye (“Biotium”, Fremont, CA, USA) for 40 min and DAPI dye (“Sigma-Aldrich”, USA) for 10 min at a concentration of 1 µg/mL. Digital images of stained cells were obtained and processed using an inverted microscope, AG Axio Observer D1, with an AxioCam HRc camera and ZEN software v3.0 (“Carl Zeiss Microscopy GmbH”, Jena, Germany).

### 2.10. GSK3β Gene Knockdown and Osteogenic Differentiation of hAD-MSCs

In order for the knockdown of the gene *GSK3β* and osteogenic differentiation induction, hAD-MSCs were incubated in the presence of collagen–fibronectin hydrogel impregnated with polyplexes containing 1.5 pmol/μL of siRNA GSK3β and PEI in a 1:3 (µg siRNA: µg PEI) ratio. The incubation of the material was carried out when it reached a monolayer confluency of 70–80% in Transwell^®^ 24-well permeable support with a pore diameter of 8 microns (Corning^®^ HTS Transwell^®^, USA) in 1 mL of Opti-MEM medium with 5% FBS (“PAA Laboratories”, USA) for 24 h.

hAD-MSCs and hAD-MSCs transfected with an equivalent amount of siRNA-C were used as negative controls, and hAD-MSCs transfected with an equivalent amount of siRNA GSK3β in the same culture plate were used as positive controls. The culture medium was replaced immediately after transfection and every 3 days with DMEM (“PanEco”, Moscow, Russia), containing 10% FBS, 4 mM of L-glutamine (“PanEco”, Moscow, Russia) and 100 mg/L of amikacin (“Synthesis”, Russia).

With a positive result in transfection after 24 h (the efficiency of siRNA-C molecule transfection is more than 90%), the expression of the *GSK3β* gene and osteogenic differentiation marker genes were evaluated on the 2nd and 7th days of the experiment.

### 2.11. Evaluation of the Expression of the GSK3β Gene and Osteogenic Differentiation Marker Genes

Total RNA was isolated from cells using “RNeasy Plus Mini Kit” (“Quagen”, Hilden, Germany); the synthesis of the first chain of total cDNA on RNA matrix was conducted using the “RevertAid kit” (Thermo Scientific, Leipzig, Germany). A quantitative real-time PCR analysis was performed on a “CFX96 Touch™” amplifier (“Bio-Rad”, Hercules, CA, USA) using intercalating “SYBR Green I” dye (“Eurogen”, Moscow, Russia) and primers specific to the *GSK3β* gene and osteogenic differentiation marker genes: *ALPL* (alkaline phosphatase), *Runx2*, *BMP-2* (bone morphogenetic protein 2), *OCN* (osteocalcin) and *OPN* (osteopontin). Target genes of the mRNA expression level were normalized by the average expression of the reference genes of *GAPDH* (glyceraldehyde 3-phosphate dehydrogenase) and *ACTB* (β-actin) (Table A2).

### 2.12. Statistical Analysis

The statistical processing of the data and graphing was performed using GraphPad Prism 8.0.1 (GraphPad, La Jolla, CA, USA). Intergroup differences were determined using the Holm–Šidák test when comparing 3 or more groups and the unpaired Student’s *t*-test when comparing 2 groups. A *p*-value <  0.05 was considered statistically significant. Data with a normal distribution are presented as average ± SD.

## 3. Results

### 3.1. Obtaining Collagen–Fibronectin Hydrogel Impregnated with siRNA GSK3β Molecules

**Figure 1:**
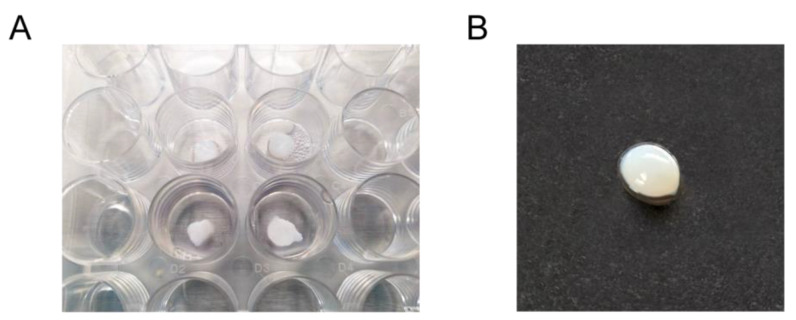
(**A**,**B**) Hardened collagen–fibronectin hydrogel impregnated with siRNA GSK3β molecules.

Osteoplastic material, based on collagen–fibronectin hydrogel and impregnated with siRNA GSK3β, was produced for the first time (Figure 1). The material is able to polymerize at 37 °C for 2 h, has a gel-like consistency in the form of a solution and good plasticity in the polymerized state. To provide osteoinductive properties to the material, siRNA GSK3β molecules in polyplexes at concentrations of 1.5 pmol/μL and 3 pmol/μL were impregnated in the collagen–fibronectin mixture. The concentrations of siRNA GSK3β molecules were calculated based on the authors’ previous studies [20].

### 3.2. Immunophenotypic Characteristics of hAD-MSCs

**Figure 2:**
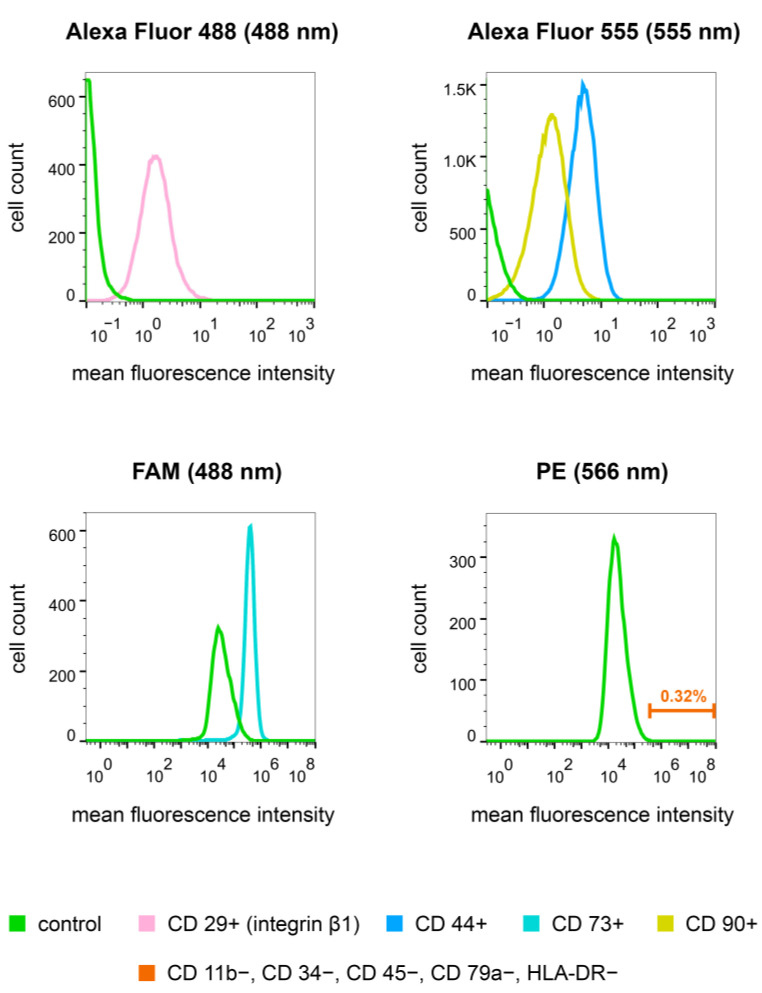
Median fluorescence intensity of mesenchymal stromal cell (MSC) markers for MSCs derived from human adipose tissue by flow cytometry.

For all cell cultures obtained from human adipose tissue, an immunophenotype corresponding to hAD-MSCs was confirmed for positive (CD29+, CD44+, CD73+ and CD90+) and negative cell-surface markers (CD11b−, CD34−, CD45−, CD79− and HLA-DR−) [21]. Below are the results of the immunophenotyping of one of the cultures (Table 3, Figure 2).

### 3.3. Investigation of the Kinetics of the Release of siRNA Molecules Impregnated in Collagen–Fibronectin Hydrogel

**Figure 3:**
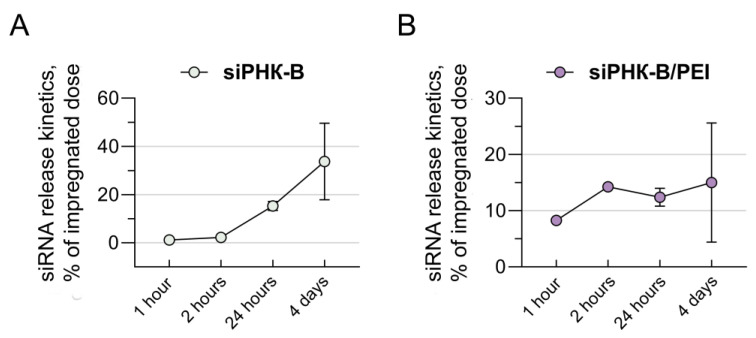
Kinetics of the release of siRNA-B molecules (**A**) and polyplexes containing siRNA-B and PEI (1:3) (**B**) from collagen–fibronectin hydrogel for 4 days using ELISA. siRNA-B—siRNA sequence-labelled biotin. PEI—polyethylenimine.

Collagen–fibronectin hydrogel provided a prolonged release of siRNA molecules during 4 days of the experiment. An evaluation of the release of 0.3 pmol/mL of siRNA-B molecules and polyplexes containing 0.3 pmol/mL of siRNA-B and PEI (1:3) from collagen–fibronectin hydrogel showed that the release from polyplexes during the first 2 h of the experiment occurs on average 6.6 times faster than the release of siRNA-B molecules without a transfection agent. Within 24 h, 1.8 times more siRNA-B molecules in polyplexes were released from collagen–fibronectin hydrogel—34.27 ± 2.5% (i.e., 1/3 of the impregnated dose) more than siRNA-B molecules without a transfection agent—18.7 ± 2.38%. After 4 days, the number of released siRNA-B molecules was equal in both experimental groups and amounted to 49.27% ± 13.12% for the group of siRNA-B molecules in polyplexes and 52.5 ± 18.3% for the group of siRNA-B molecules without a transfection agent (Figure 3). It can be concluded that the addition of PEI to siRNA-B molecules significantly accelerates their release from collagen–fibronectin hydrogel within 24 h. To ensure the concentration of siRNA molecules necessary for the effective transfection of 50 pmol/mL, it is required to increase the dose of siRNA molecules by 3–5 times when they are impregnated into collagen–fibronectin hydrogel.

It is known that in the presence of PEI, plasmid DNA and siRNA molecules condense and compact, resulting in forming polyplexes with a common positive charge, capable of interacting with negatively charged heparan sulfates and other proteoglycans on the cell surface [22,23,24,25,26]. This property of PEI may explain the faster release of polyplexes formed with its help in comparison with free monomeric siRNA-B molecules from the collagen–fibronectin hydrogel.

### 3.4. Assessment of the Transfection Efficiency of siRNA Molecules Impregnated in Collagen–Fibronectin Hydrogel

**Figure 4:**
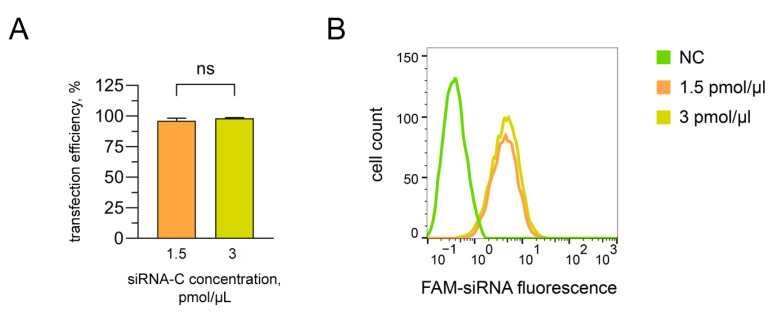
The transfection efficiency of siRNA-C molecules impregnated in collagen–fibronectin hydrogel on the 2nd day after transfection (**A**). A histogram overlay of mesenchymal stromal cell from human adipose tissue after incubation with 1.5 and 3 pmol/µL siRNA-C, respectively (**B**). Flow cytometry. ns—not significant. NC—control.

The transfection efficiency of hAD-MSCs was 96.08 ± 2.25% and 98.19 ± 0.54%, respectively, during an incubation with hydrogels containing 1.5 and 3 pmol/µL siRNA-C. No statistically significant differences were detected when using these concentrations (Figure 4).

For further experimental studies with the aim of impregnation in collagen–fibronectin hydrogel, a lower effective concentration of siRNA was chosen—1.5 pmol/μL.

### 3.5. Cytotoxic Effect of Collagen–Fibronectin Hydrogel Impregnated with siRNA GSK3β Molecules

**Figure 5:**
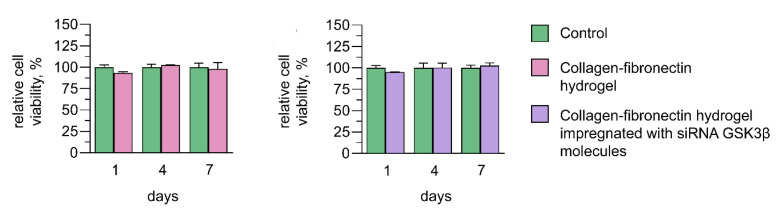
Cytotoxic effect of collagen–fibronectin hydrogel and collagen–fibronectin hydrogel impregnated with polyplexes containing 1.5 pmol/μL siRNA GSK3β molecules and PEI (1:3) on days 1, 4 and 7 using MTT assay.

Collagen–fibronectin hydrogel separately and impregnated with 1.5 pmol/μL siRNA GSK3β molecules had no cytotoxic effect and maintained hAD-MSC adhesion. Cell viability in the presence of collagen–fibronectin hydrogel did not significantly differ from the values of the control group and amounted to 93.31 ± 1.59% on day 1, 102.37 ± 0.68% on day 4 and 97.99 ± 7.52% on day 7 of the experiment. Cell viability in the presence of collagen–fibronectin hydrogel impregnated with siRNA GSK3β also did not significantly differ from the values of the control group and amounted 95.06 ± 0.17% on day 1, 100.27 ± 5.23% on day 4 and 102.69 ± 3.13% on day 7 of the experiment (Figure 5).

**Figure 6:**
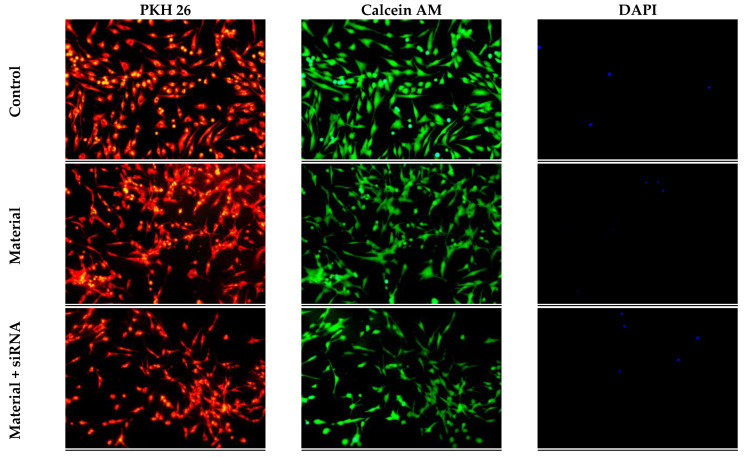
Adhesion of mesenchymal stem cells derived from human adipose tissue on surface of the collagen–fibronectin hydrogel impregnated with 1.5 pmol/μL siRNA GSK3β molecules (on the 2nd day after impregnation). Stained for PKH-26, Calcein AM and DAPI using fluorescent microscopy with 100× magnification.

Most of the cells placed on the material acquired a spindle-shaped morphology and continued to be vital after a day (Figure 6). Thus, the surface structure of collagen-fibronectin hydrogel separately and impregnated with polyplexes containing 1.5 pmol/µL siRNA GSK3β-3 molecules is biocompatible and promotes cell adhesion.

On day 2 after incubation with collagen–fibronectin hydrogel impregnated with siRNA GSK3β molecules, the expression of *GSK3β* gene decreased by 63.3 ± 1.75%.

The expression of *Runx2* gene in the presence of collagen–fibronectin hydrogel impregnated with siRNA GSK3β molecules increased by 1.6 ± 0.3 times (*p* = 0.0392).

**Figure 7:**
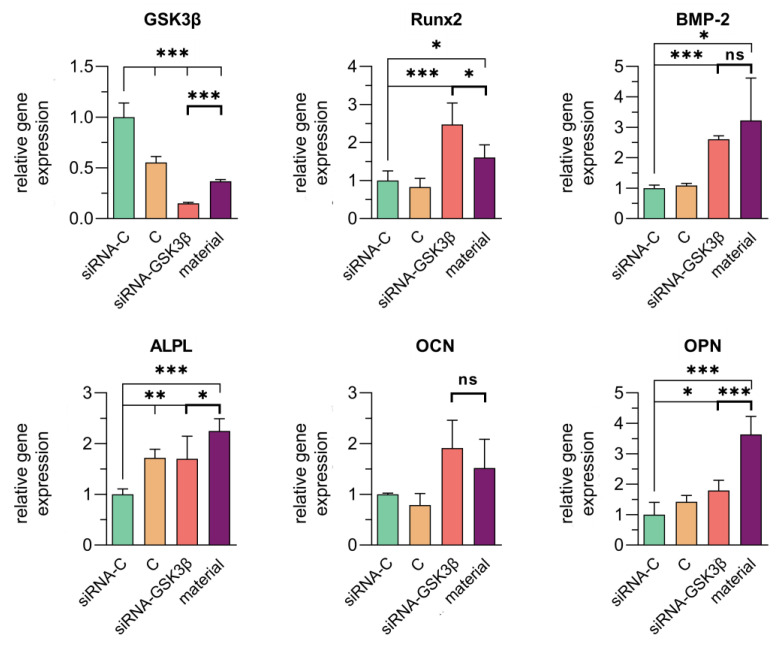
Expression of the *GSK3β* gene and osteogenic differentiation marker genes (*Runx2*, *BMP-2*, *ALPL*, *OCN* and *OPN*) in human mesenchymal stem cells derived from human adipose tissue cultures (hAD-MSCs) on day 2 after incubation with collagen–fibronectin hydrogel impregnated with polyplexes containing 1.5 pmol/μL of siRNA GSK3β and PEI (1:3) using quantitative real-time PCR analysis. Significance of statistical differences for the experimental groups relative to controls at *p* ≤ 0.05 is designated as “*”, *p* < 0.01—as “**” and *p* < 0.001—as “***”; ns—not significant (*p* > 0.5). siRNA-C—сontrol siRNA-FAM incubated with hAD-MSCs. C—control (only hAD-MSCs). siRNA GSK3β—hAD-MSCs incubated with siRNA GSK3β. Material—collagen–fibronectin hydrogel incubated with hAD-MSCs and impregnated with siRNA GSK3β molecules.

The incubation of hAD-MSCs with collagen–fibronectin hydrogel impregnated with siRNA GSK3β molecules also led to an increase in the expression of osteogenic differentiation marker genes: *ALPL* by 2.3 ± 0.2 times (*p* < 0.0001), *BMP-2* by 3.2 ± 1.4 times (*p* = 0.0136), *OCN* by 1.5 ± 0.6 times (no significant) and *OPN* by 3.6 ± 0.6 times (*p* < 0.0001) (Figure 7).

On day 7 after incubation with collagen–fibronectin hydrogel impregnated with siRNA GSK3β molecules, the expression of *GSK3β* gene remained at the level of 37.8 ± 4.32%. The expression of the *Runx2* gene increased by 1.6 ± 0.5 times.

**Figure 8:**
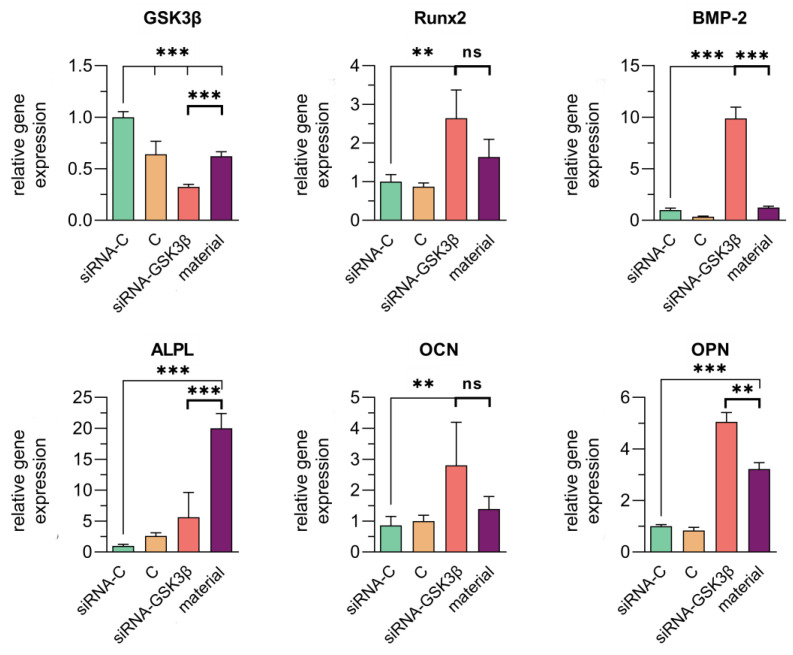
Expression of the *GSK3β* gene and osteogenic differentiation marker genes in mesenchymal stem cells derived from human adipose tissue (hAD-MSCs) cultures on day 7 after incubation with collagen–fibronectin hydrogel impregnated with polyplexes containing 1.5 pmol/μL of siRNA GSK3β and PEI (1:3) using quantitative real-time PCR analysis. Significance of statistical differences for the experimental groups relative to controls at *p* < 0.01 is designated as “**” and *p* < 0.001—as “***”; ns—not significant (*p* > 0.5). siRNA-C—сontrol siRNA-FAM incubated with hAD-MSCs. C—control (only hAD-MSCs). siRNA GSK3β—hAD-MSCs incubated with siRNA GSK3β. Material—collagen–fibronectin hydrogel incubated with hAD-MSCs and impregnated with siRNA GSK3β molecules.

The incubation of hAD-MSCs with collagen–fibronectin hydrogel impregnated with siRNA GSK3β molecules again led to an increase in the expression of gene *ALPL* by 20 ± 2.4 times (*p* < 0.0001), *BMP-2* by 1.2 ± 0.1 times (no significant), *OCN* by 1.4 ± 0.4 times (no significant) and *OPN* by 3.2 ± 0.3 times (*p* < 0.0001) (Figure 8).

Compared to the previous term, the expression of the *Runx2* gene increased by an average of 3.4 times and the expression of the remaining osteogenic differentiation marker genes increased 2–8-fold and 1.5–3-fold.

Thus, the efficiency of osteogenic differentiation in hAD-MSCs cultures can be increased by a knockdown of the *GSK3β* gene using gene-targeted siRNA molecules. The osteoinducing effect of collagen–fibronectin hydrogel impregnated with siRNA GSK3β at a concentration of 1.5 pmol/µL was observed, which indicates the preservation of functional activity of siRNA GSK3β molecules in the osteoplastic material.

## 4. Discussion

Osteogenesis is a dynamic and complex process that begins as early as in the first month of fetal development. Its regulation involves many proteins included in different signaling pathways, including Wnt/β-catenin and BMP/SMAD, as well as TGF-β, which activates transcription factors Runx2 and Osterix, insulin/PI3K/Akt, Hedhehog and many others [27].

The inhibition of *GSK3β* in vivo enhances the cartilage degeneration in a mouse model of posttraumatic osteoarthritis. This is due to an increase in the expression of MMP-10 and MMP-14 (the main collagenase activators) and a decrease in TIMP-3 (the pivotal inhibitor of both collagenases and aggrecanases) [28]. The consequence of inhibition of *GSK3β* by small molecule CHIR99021 is the intracellular accumulation of β-catenin, where it induces the gene transduction important for osteoblast differentiation, while at the same time suppresses the chondrocyte differentiation [29]. The *GSK3* knockout leads to a serious decrease in the growth of the endochondral bone, as well as the premature remodeling of the growth plate. Articular cartilage remains unchanged at the same time [30].

It is also known that the inhibition of *GSK3β* leads to an increase in the expression of osteogenic *Alp*, *Runx2*, *Ocn* and *Col1a1* [31]. One of these inhibitors is LiCl, which, by activating Wnt signaling, promotes osteoblastogenesis and reduces adipogenesis [32]. The administration of LiCl increased the bone mass in normal C57BL/6 mice and in SAMP6 osteoporosis model mice. The other small molecule, AR28 inhibitor is able to induce the nuclear translocation of β-catenin and increase bone mass. This is the result of the early activation of MSCs with osteogenic and adipogenic potential, but the osteoblast differentiation is due to adipogenesis [33]. The treatment of human osteoblast cells with small molecule *GSK3* inhibitor AZD2858 increases the level of β-catenin after a short period of time. This drug may be effective as an anabolic strategy, since it has extensive bone-forming effects in predominantly trabecular areas [34]. Fracture healing in rats injected with AZD2858 occurred faster, but they did not have a preliminary formation of cartilage tissue. This was due to the fact that mesenchymal cells immediately differentiated into osteoblasts [35]. Stem cells derived from adipose tissue also have gone along the osteoblastogenesis path after treatment with AZD2858, as well as AR79 and A13282107 small molecule inhibitors. On the 14th day of therapy, bone mineral density and trabecular thickness increased [36]. In a model of collagen-induced rheumatoid arthritis, the small molecule *GSK3β* inhibitor TDZD-8 reduced bone resorption [37]. This was associated with the suppression of microRNA-155 and microRNA-24 expression [38].

Prior studies have implicated the contribution of collagen and fibronectin to the proliferation of MSCs, their survival under stress and their good cell adhesion on the surface of such materials, as well as their osteogenic differentiation [39]. Even collagen materials of a xenogeneic origin demonstrate a high biocompatibility [40,41].

In this work, using MTT assay, the great cytocompatibility of the collagen–fibronectin hydrogel separately impregnated with polyplexes containing siRNA GSK3β molecules was detected. The ability of materials to adhere cells on their surface is extremely important and a fundamental condition for their subsequent proliferation, migration and differentiation [42]. The first and most significant phase of bone tissue regeneration is osteoconductive. The phenomenon of osteoconduction is based on the attraction and migration of osteogenic cells to the surface of the material, as well as their adhesion, proliferation and further formation of ECM [43,44]. Materials that promote the specific adhesion of osteoblasts can increase the efficiency of their differentiation, contributing to the mineralization of ECM and an increase in the volume of newly formed bone tissue [45]. By staining cells with PKH-26, Calcein AM, DAPI and fluorescence microscopy, it was shown that collagen–fibronectin hydrogel, separately impregnated with polyplexes containing siRNA GSK3β molecules, effectively maintains cell adhesion on its surface.

Previously, biomaterials impregnated with siRNA molecules aimed at osteogenic differentiation inhibitor genes were described in several papers, such as the article by Noggin et al. The use of such biomaterials led to the effective knockdown of target genes, induction of osteogenic differentiation and reduction of bone tissue regeneration time in vitro and in vivo models, respectively [46,47,48]. Furthermore, the incubation of hAD-MSCs with siRNA GSK3β molecules led to an increase in the expression of osteogenic markers RUNX2, OSX, SATB2, BSP, OPN and OCN at the level of mRNA and protein [10].

## 5. Conclusions

We have developed an osteoplastic material based on collagen–fibronectin hydrogel impregnated with siRNA GSK3β molecules; its osteoinducing effect has been confirmed in in vitro experiments. The material is capable of thermal curing at 37 °C for 2 h, and it has a gel-like consistency in the uncured state and a good plasticity in the hardened state. pH values of the material at the final stages of the thermal curing process were 7.3–7.4, which correspond to the pH of the internal environment of the human body. It is shown that all components and the integral composition of the osteoplastic material have a high biocompatibility. A high ability of adhesion and cell proliferation on the surface of the material has been demonstrated, which is important for ensuring its osteoconductive properties. In the prospect, the material can be recommended for use in the field of traumatology, dentistry, maxillofacial surgery and orthopedics.

## Figures and Tables

**Table 1:** Sequences of siRNA molecules.

Name	Strand	Sequence in 5′ → 3′ Direction
Control siRNA-FAM (siRNA-C)	Sense	FAM-AGGUCGAACUACGGGUCAAdTdC
	Antisense	FAM-UUGACCCGUAGUUCGACCUdAdG
Control siRNA-biotin (siRNA-B)	Sense	biotin-AGGUCGAACUACGGGUCAAdTdC
	Antisense	biotin-UUGACCCGUAGUUCGACCUdAdG
siRNA GSK3β	Sense	CCAACAAGGGAGCAAAUCAGAdTdT
	Antisense	UCUGAUUUGCUCCCUUGUUGGdTdT

**Table 2:** Quantities of siRNA and transfection agent molecules based on 1 sample as per the material.

Reagent	Quantities	
Volume of siRNA, µg	0.67	0.8
Amount of siRNA, pmol	50	60
Amount of PEI at a ratio of 1:3, µg	2	2.4
Volume of DPBS for dilution of siRNA, μL	up to 25	up to 25
Volume of DPBS for dilution of PEI, μL	up to 25	up to 25

**Table 3:** Percentage values of the immunophenotypic characterization of mesenchymal stem cells derived from human adipose tissue (hAD-MSCs) by flow cytometry.

Positive and Negative CD Markers of hAD-MSCs	The Relative Number of Cells in the Culture
CD29+ (integrin β1)	99.65%
CD44+	99.93%
CD73+	75.4%
CD90+	80.38%
CD11b−, CD34−, CD45−, CD79a−, HLA-DR−	0.32%

## Data Availability

The datasets used and/or analyzed during the current study are available from the corresponding author on reasonable request.

## Abbreviations

6-FAM: 6-carboxyfluorescein; ACTB: actin beta; ALPL: alkaline phosphatase; BMP-2: human bone morphogenetic protein-2; DMEM: Dulbecco’s modified Eagle’s medium; DPBS: Dulbecco’s phosphate-buffered saline; FBS: fetal bovine serum; ECM: extracellular matrix; ELISA: enzyme-linked immunosorbent assay; GAPDH: glyceraldehyde 3-phosphate dehydrogenase; GSK3β: glycogen synthase kinase-3β; hAD-MSCs: human adipose tissue-derived mesenchymal stem cells; MSCs: mesenchymal stem cells; OCN: osteocalcin; OPN: osteopontin; PAGE: polyacrylamide gel electrophoresis; PEI: polyethylenimine; PCR: polymerase chain reaction; Runx2: runt-related transcription factor 2; SC-siRNA: scrambled siRNA sequence labelled by 6-carboxyfluorescein; siRNA: small interfering RNA; siRNA-B: siRNA sequence labelled biotin; siRNA-C: siRNA sequence labelled by 6-carboxyfluorescein.

## Appendix A

**Table A1:** Positive and negative CD markers of cell surface of mesenchymal stem cells derived from human adipose tissue (hAD-MSCs).

Positive CD Markers of hAD-MSCs	Negative CD Markers of hAD-MSCs
CD 29+ (integrin β1), rabbit IgG, clone EP1041Y	CD 11b− PE, mouse IgG2B; clone 238446
CD 44+, mouse IgG2a, clone F10-44-2	CD 34− PE, mouse IgG1, clone QBEnd10
CD 90+ (Thy1), mouse IgG1, clone AF-9	CD 45− PE, mouse IgG1, clone 2D1
CD73+ FAM, mouse IgG2B, clone 606112	CD 79a− PE, mouse IgG1, clone 706931
	HLA-DR− PE, mouse IgG1, clone L203

**Table A2:** Gene-specific primers’ sequences.

Name	Sequence in 5′ → 3′ Direction
*ACTB* forward	CCTGGAACCAGCACAATA
*ACTB* reverse	GGGCCGGACTAGTCATAC
*ALPL* forward	CTCGGAAGACACTCTGACCGT
*ALPL* reverse	TAGTCCACCATGGAGACATTCTCT
*BMP-2* forward	ACTACCAGAAACGAGTGGGAA
*BMP-2* reverse	GCATCTGTTCTCGGAAAACCT
*GAPDH* forward	GAAGGTGAAGGTCGGAGTACA
*GAPDH* reverse	TTCACACCCATGACGAGACAT
*GSK3β* forward	ACGGCACCCAAATATCAAACT
*GSK3β* reverse	AGCCAGAGGTGGATTACTTGA
*OCN* forward	CAGAGTCCAGCAAAGGTGCAG
*OCN* reverse	CTCCCAGCCATTGATACAGGT
*OPN* forward	GCCGAGGTGATAGTGTGGTT
*OPN* reverse	AACGGGGATGGCCTTGTATG
*Runx2* forward	AGTGGACGACAGTCTGACTTT
*Runx2* reverse	GGTGAATGCGGTAAGACTGGT
